# Retinoic acid inhibits the cytoproliferative response to weak 50-Hz magnetic fields in neuroblastoma cells

**DOI:** 10.3892/or.2012.2212

**Published:** 2012-12-24

**Authors:** MARÍA ÁNGELES TRILLO, MARÍA ANTONIA MARTÍNEZ, MARÍA ANTONIA CID, ALEJANDRO ÚBEDA

**Affiliations:** Department of Research-BEM, IRYCIS, Hospital Ramon y Cajal, 28034 Madrid, Spain

**Keywords:** power frequency, extremely low frequency, all-*trans*-retinoic acid, NB69, proliferation, proliferating cell nuclear antigen

## Abstract

We previously reported that intermittent exposure to a 50-Hz magnetic field (MF) at 100 μT stimulates cell proliferation in the human neuroblastoma cell line NB69. The present study aimed to investigate whether the magnetic field-induced growth promotion also occurs at a lower magnetic flux density of 10 μT. To this purpose, NB69 cells were subjected for 42 h to intermittent exposure, 3 h on/3 h off, to a 50-Hz MF at a 10 or 100 μT magnetic flux density. The field exposure took place either in the presence or in the absence of the antiproliferative agent retinoic acid. At the end of the treatment and/or incubation period, the cell growth was estimated by hemocytometric counting and spectrophotometric analysis of total protein and DNA contents. Potential changes in DNA synthesis were also assessed through proliferating cell nuclear antigen (PCNA) immunolabeling. The results confirmed previously reported data that a 42-h exposure to a 50-Hz sine wave MF at 100 μT promotes cell growth in the NB69 cell line, and showed that 10 μT induces a similar proliferative response. This effect, which was significantly associated and linearly correlated with PCNA expression, was abolished by the presence of retinoic acid in the culture medium.

## Introduction

A number of epidemiological studies have provided indications that workers chronically exposed to medium-to-high levels of extremely low frequency (ELF) magnetic fields (MFs), including those of a power frequency 50–60 Hz have an increased risk of developing various types of cancer (1–3) or degenerative diseases (4,5), although other studies have not detected such increases in risk (6,7). The International Agency for Research on Cancer (IARC) has classified ELF MF exposure as a ‘possible carcinogen to humans’ (8) on the basis of the epidemiological and experimental evidence, particularly on adult and childhood leukemia. Nevertheless, uncertainty persists on this matter, and other international bodies, including the International Commission on Non-Ionizing Radiation Protection (ICNIRP), consider that the current evidence on the carcinogenic potential of weak ELF fields (B≤0.5 mT at 50 Hz) is insufficient, primarily due to the present lack of a mechanistic basis that can explain the phenomena underlying the biological interactions of those fields (9,10). Experimental studies *in vivo* have yielded inconclusive or contradictory results. The lack of properly standardized exposure parameters may have contributed to such negative or inconsistent results (11–13). It should, moreover, be considered that animal models may be unsuitable for studies examining various aspects of the carcinogenic potential of weak ELF fields (14). As for the cellular response, a large number of studies have reported *in vitro* effects of an ELF-MF on different cellular processes in different cell species (reviewed in ref. 15). However, to date there is no general agreement on the specific primary biological targets of power frequency MFs, on the biophysical and chemical basis of the MF interactions, nor to which extent the reported *in vitro* bioeffects may be indicative of a human susceptibility to these fields. Concerning potential targets related to cancer risk, ELF MFs have been shown to alter gene and protein expression (16–18) as well as intracellular calcium concentration and apoptosis rates (19–23) and to induce oxidative stress (24–26) and DNA damage (27–31). The interest in the potential adverse effects of power frequency MFs has also been focused on the possibility that these fields may influence tumor promotion by increasing the rate of cell proliferation and/or modifying the activity of molecules implicated in their regulation. However, the number of studies examining these processes under exposure to low magnetic flux densities, B≤100 μT, which can be found in certain occupational environments, is rather scarce. On the other hand, it has been described that the cellular alterations due to ELF-MF exposure, combined with certain risk factors and/or in combination with the action of other physiological or environmental agents, may influence tumorigenic processes (32,33).

In this respect, we previously reported that a 50-Hz MF at 100 μT significantly increased the cell number and BrdU incorporation into DNA in the human cell lines NB69 (neuroblastoma) and HepG2 (hepatocarcinoma). These effects were modulated by all-*trans* retinol, the metabolic precursor of retinoic acid (34). The present study assessed the responsiveness of the NB69 cell line exposed to a 50-Hz MF at 10 and 100 μT in combination with the retinoid retinoic acid (RA), in regards to cell proliferation. RA plays a crucial role in the growth and differentiation of normal, premalignant and malignant tissues, and has received significant attention because of its potential interest in cancer therapy (35–37). RA is currently being used as a tool in standard treatment protocols for high-risk neuroblastomas, and is one of the well-established inducers of neuronal differentiation and/or apoptosis in neuroblastoma cells (38–40). For example, in the neuroblastoma cell line LAN-5, exposure to a 50-Hz MF at 1 mT was reported to exert an antagonistic effect on cell differentiation induced by 5 μM RA (24). The flux density of 10 μT tested in the present study, is found in certain occupational environments (41), corresponds to 10 and 2%, respectively, of the reference levels proposed by the ICNIRP for the protection of the general public (100 μT) and workers (500 μT) against the harmful effects of short-term exposure to a 50-Hz MF (9,10). In the present study, as an initial step to determine whether an MF at 10 μT induces proliferative effects similar to those we reported at 100 μT, the potential changes in cell viability and proliferation were evaluated by cell counting (trypan blue exclusion), by spectrophotometric analysis of total protein and DNA content and by the immunocytochemical expression of the proliferative marker, proliferating cell nuclear antigen (PCNA). Subsequently, the responsiveness of the NB69 cell line to MF at 10 and 100 μT when in the presence of RA was assessed in regards to cell viability and proliferation.

## Materials and methods

### Magnetic field exposure

Fifty-hertz, sine wave magnetic fields at 10 or 100 μT were generated by a set of coil pairs in a Helmholtz configuration and energized using a Newtronics Model 200MSTPC wave generator (Madrid, Spain). The exposure set-up used in these experiments was based on that developed by Blackman *et al*(42), and has been described elsewhere (34). Briefly, each of the two identical sets consisted of a pair of 1000-turn, 20-cm diameter coils of enamelled copper wire, aligned coaxially 10 cm apart and oriented to produce vertically polarized magnetic fields. Five or twenty 60-mm Petri dishes (Nunc, Spain) were placed in the uniform MF space within the coils for exposure or sham-exposure. Currents in the coils were adjusted and monitored using a multimeter (model 974A; Hewlett Packard, Loveland, CO, USA) after establishing the DC and AC flux densities with fluxgate magnetometers (Bartington model Mag-3; GMW Associates, San Carlos, CA, USA and EFA-3 model BN 2245/90.20; Wandel & Goltermann S.A, Eningen, Germany). The coil sets were mounted in the center of magnetically shielded, Co-Netic alloy boxes (Amuneal Corp., Philadelphia, PA, USA) housed in incubators (Forma models 3121 and 3194) in an atmosphere of 5% CO_2_ at 37°C. The magnetic shielding allowed for reduced environmental fields at the sample locations, with DC MF=0.02–0.08 μT (rms) and 50 Hz AC MF=0.06–0.09 μT (rms). Two identical sets of coils, shielding rooms and incubators were used. In each experimental run, only one set of coils was energized at random. The samples in the unenergized set were considered sham-exposed controls. The exposure settings and the dosimetry were assessed and validated immediately before and after each experimental run.

### Cell culture

The NB69 human neuroblastoma cell line was provided by Dr M.A. Mena (Hospital Ramón y Cajal, Madrid, Spain). The cells were plated at a density of 4.5×10^4^ cells/ml in Petri dishes and grown in Dulbecco’s minimum Essential medium (DMEM; Gibco, Carlsbad, CA, USA) supplemented with 15% heat inactivated fetal calf serum (FCS, Gibco), 4 mM L-glutamine and 100 U/ml penicillin plus 100 U/ml streptomycin. In each experimental run, cells seeded in groups of 10 or 40 dishes were grown for three days in humidified incubators with an atmosphere of 5% CO_2_ at 37°C. When appropriate, the medium was supplemented at plating with 2 μM RA (all-*trans*-retinol; Worthington Biochemical Corp., Lakewood, NJ, USA), or with the corresponding vehicle, absolute ethanol (controls). At day 3 post-plating the cultures received fresh medium supplemented with a second dose of 2 μM RA or with the matched vehicle concentration.

### Assessment of cellular response to RA

Cells were seeded and supplemented with RA concentrations within the physiological range in mammals: 0.5, 1.0, 2.0 or 5.0 μM. RA untreated controls, supplemented with the highest vehicle concentration used in these experiments, were also included in each run. Seventy-two hours after plating, the medium was renewed and supplemented with the corresponding concentrations of RA or vehicle. Each concentration was quadruplicate tested (a total of 24 Petri dishes/experimental replicate). A total of 3 experimental replicates were carried out. At the end of five days of incubation in the presence of RA or vehicle, the cells were collected, counted and analyzed for cell viability and proliferation.

### Exposure to a 50-Hz MF

Two series of experiments were conducted. In the first series, a total of 10 experimental replicates at a magnetic flux density B=10 μT, and 5 replicates at 100 μT, were carried out. In each experimental replicate 10 dishes with cells were grown for 3 days inside a commercial incubator. On day 3, the medium was renewed and each group of 5 dishes was transferred to one of two identical incubators, (atmosphere of 5% CO_2_ at 37°C and 100% humidity) with the shielded chambers and Helmholtz coils inside, as previously described. These incubators were used for MF-exposure or sham-exposure in alternating runs. The MF-treated groups were exposed to the fields intermittently, 3 h on/3 h off, for 42 h.

In the second experimental series, a total of 9 replicates, 4 at B=10 μT and 5 at 100 μT, were carried out. In each replicate a total of 40 dishes (20 dishes for the 42-h studies and 20 for the 90-h studies) was distributed in groups of 5 Petri dishes and exposed or sham-exposed to RA and/or MF, according to the following combinations: MF−/RA-, MF−/RA+, MF+/RA- and MF+/RA+. The MF and RA treatments, as well as the respective sham-treatments, were applied according to the procedures previously described. At the end of the 42-h or 90-h exposure and/or incubation, the cell growth and viability of the NB69 cells were determined using a hemocytometer. Spectrophotometric analysis of total protein and DNA contents were conducted following the methods described below.

### Cell counting and spectrophotometric analysis of protein and DNA contents

In all experiments the MF was applied at day 3 post-plating. The cell counting was performed at the end of 42 h or 90 h of treatment and/or incubation, which corresponds to days 5 or 7 post-plating, respectively. At the end of these periods the cells were scrape-detached from the culture dishes, pipette-disaggregated, collected and divided in aliquots. The total cell number and viability were determined by trypan blue exclusion and each sample was double-counted in a manner blinded to the treatment method. The remaining aliquots were used for spectrophotometric quantification of protein and DNA contents. Protein content was determined using Bradford’s technique (43), using bovine serum albumin as a standard. For DNA quantification, the Burton’s method (44) was applied, using 2-deoxy-D-ribose (Sigma, Steinheim, Germany) as standard.

### Immunocytochemical analysis of PCNA

Proliferating cell nuclear antigen (PCNA), the auxiliary component of DNA polymerase δ, is a 36-kDa nuclear protein synthesized in the late G1 and S phases of the cell cycle, and constitutes a useful proliferation marker (45,46). Cell samples were seeded on coverslips placed at the bottom of 60-mm Petri dishes (n=2 coverslips per dish; 8 dishes per experimental replicate), incubated for three days and exposed intermittently for an additional 42 h to a 50-Hz MF at 10 or 100 μT. In the experiments at 10 μT, additional samples were seeded (n=8 dishes per experimental replicate) for cell counting by trypan blue exclusion. PCNA expression was analyzed through indirect immunofluorescence. For this purpose, at the end of the exposure and/or incubation interval, cells on coverslips were incubated with the monoclonal antibody anti-PCNA (FL-261; Santa Cruz Biotechnology, Quimigen S.L., Madrid, Spain) and with an Alexa Fluor Green secondary antibody (Molecular Probes, Invitrogen, Prat de Llobregat, Barcelona, Spain). Hoechst 33342 was added to the mounting medium as a counterstain for nuclei. Background controls without the primary antibody were also analyzed. The samples were evaluated by a photomicroscope (Nikon Eclipse TE300; Melville, NY, USA) and computer-assisted image analysis (AnalySIS: Soft Imaging Systems GmbH, Munich, Germany). In each of a total of 8 experimental replicates, 4 dishes were studied per experimental condition, MF or sham exposure. Twenty random microscopic fields per coverslip were evaluated, and ~5,000 cells were studied per replicate and experimental group. The percentage of PCNA-positive cells was calculated against the total number of cells.

### Statistical analysis

All experimental and analytical procedures were conducted in a blinded manner to the treatment method. The data were normalized over the respective control samples, and the values were presented as the means ± SEM of at least three independent experimental replicates. The data corresponding to treated groups and their respective controls were compared by the two-sample Student’s t-test. The multifactorial one-way analysis of variance, ANOVA, was used to assess differences between multiple sets of data. The limit of statistical significance was set at P<0.05.

## Results

### First experimental series: cell growth and viability after MF exposure

**Figure 1:**
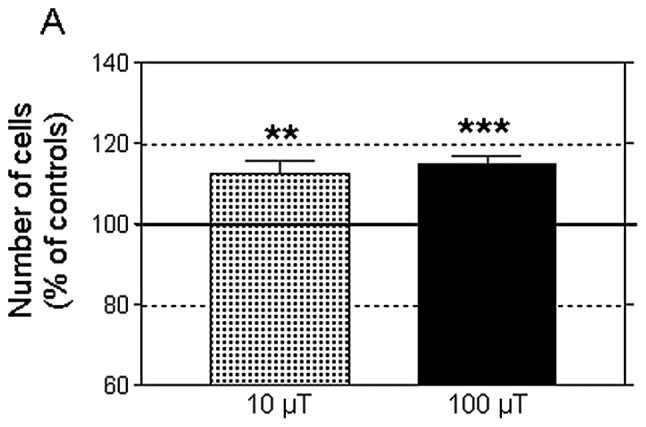

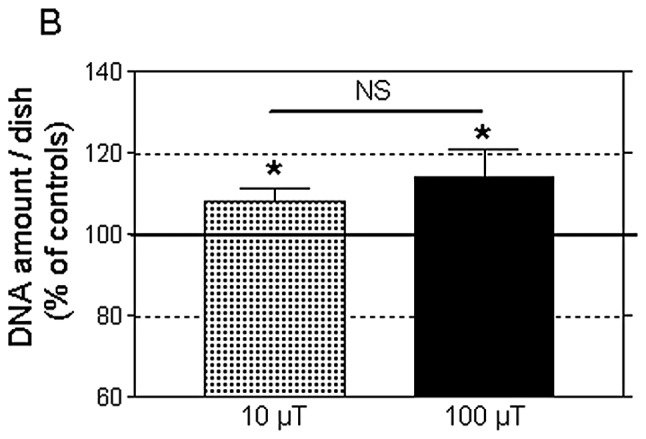
Effect of 42 h of intermittent exposure (3 h on/3 h off) to MF at 10 or 100 μT on, (A) cell number (B) DNA content (mg/dish). Means ± SEM of 10 and 5 experimental replicates at 10 and 100 μT, respectively; normalized values over the respective sham-exposed controls (5 MF-exposed dishes per replicate). NS, not significant; ^*^P<0.05, ^**^P<0.01, ^***^P<0.001 (ANOVA followed by unpaired Student’s t-test).

A 42-h intermittent exposure to an MF at 10 or 100 μT significantly increased the average number of NB69 cells when compared to the corresponding controls (12.5 and 14.8%, respectively, Fig. 1A). The MF treatments did not influence cell viability, which was ~85%, both in the MF-treated and in the sham-exposed samples. Nor were significant changes observed in the percentages of necrotic cells after exposure to 10 or 100 μT MF (2 and 4.5% over the controls, respectively) as analyzed by trypan blue dye exclusion staining. The total protein content, quantified through spectrophotometric analysis, revealed no differences between samples exposed to MF at 10 or 100 μT and their controls (data not shown). However, the DNA content was significantly increased by 8.1 and 17.4% over the controls at 10 and 100 μT MF, respectively (Fig. 1B). As a whole, these results confirmed previously reported findings that intermittent exposure to 50-Hz MF stimulates cell growth in human neuroblastoma cultures (34,47) and showed that an MF at 10 and 100 μT induces equivalent proliferative responses.

### Second experimental series: cell growth response to combined treatment with MF and retinoic acid

**Figure 2:**
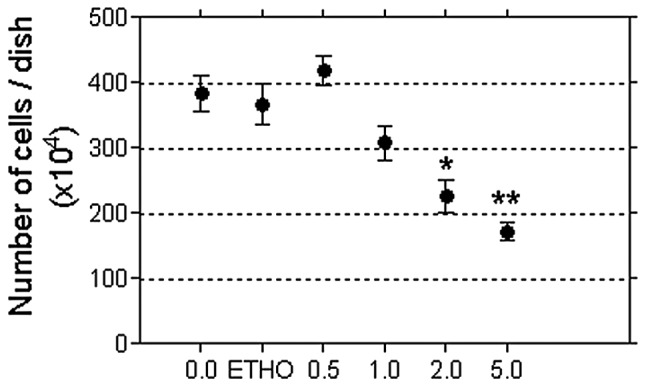
Number of cells at the end of day 5 following treatment with different RA concentrations (0.5, 1.0, 2.0 and 5.0 μM). Each data point represents a total of 12 dishes from 3 independent replicates per concentration. The vehicle, ethanol (ETHO), at the highest concentration tested (corresponding to 5.0 μM RA) did not influence cell growth when compared to the controls without vehicle (0.0). ^*^P≤0.05, ^**^P≤0.01 (ANOVA followed by unpaired Student’s t-test). A significant negative correlation between both variables, the RA concentration and the cell number, was observed (Pearson’s r=−0.8915; P<0.05).

Fig. 2 shows the results of a pilot study testing the NB69 cell growth response to 5 days of treatment with RA at concentrations of 0.5 to 5.0 μM, which fall within the physiological range in mammals. RA induced a linear, dose-dependent decrease in cell number (Pearson’s correlation coefficient, r=−0.8915; P<0.05). Based on this result, the intermediate dose of 2 μM RA was selected to investigate the influence of MF on the cell growth response to RA.

**Figure 3:**
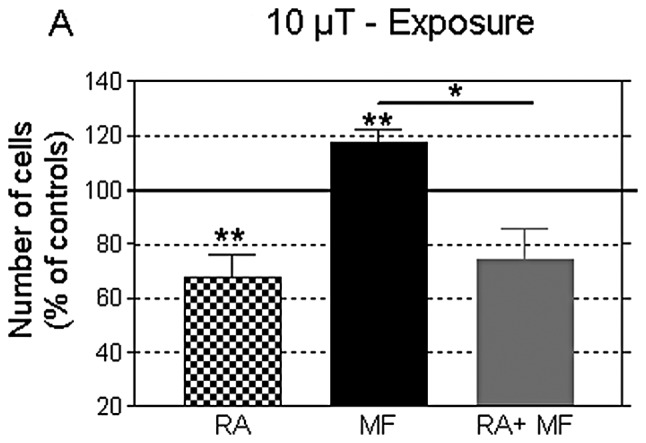

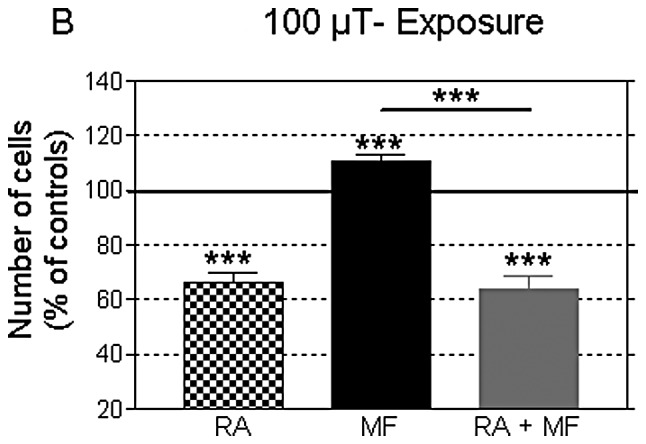
Cell number at the end of day 5 following the different testing conditions: control (sham), RA, MF and combined treatment with RA+MF. The MF-exposed group was treated for 42 h, starting at day 3 post-plating. In RA-treated groups the medium was supplemented at days 0 and 3 post-plating with 2 μM RA. (A) Exposure to 10 μT; n=4 experimental replicates; 20 dishes per replicate and experimental condition. (B) Exposure to 100 μT; n=5 experimental replicates; 20 dishes per replicate and 25 dishes per condition. Data were normalized according to the values of the controls, sham-exposed to MF+0.0 μM RA, and expressed as the means ± SEM. ^*^P≤0.05, ^**^P≤0.01, ^***^P≤0.001 (ANOVA followed by unpaired Student’s t-test).

As shown in Fig. 3, at the end of day 5 post-plating, a 42-h exposure to MF at 10 or 100 μT induced significant increases in cell growth (17.7 and 11.0% over controls; P<0.01 and P<0.001, respectively), confirming the results obtained in the first experimental series. In turn, this growth-promoting effect elicited by the MFs was fully blocked by the presence of 2 μM RA in the culture medium. Furthermore, the cell number at the end of the combined treatment was significantly lower than that in the controls, and equivalent to that in samples treated with RA only. The cell viability was not significantly altered in any of the conditions tested (data not shown). These results indicated that RA caused NB69 cells to be irresponsive or insensitive to the MF-induced cytoproliferative effects.

**Figure 4:**
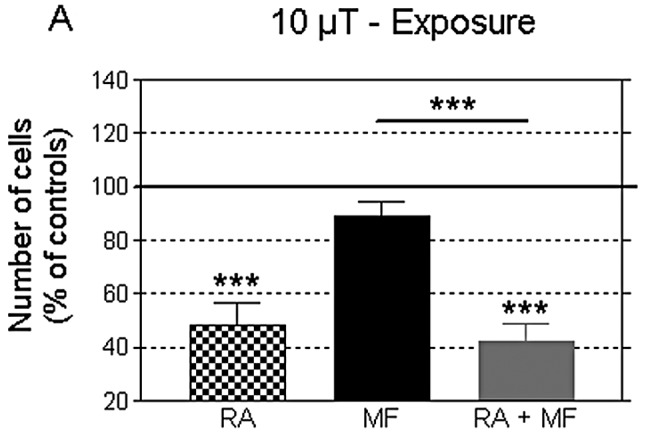

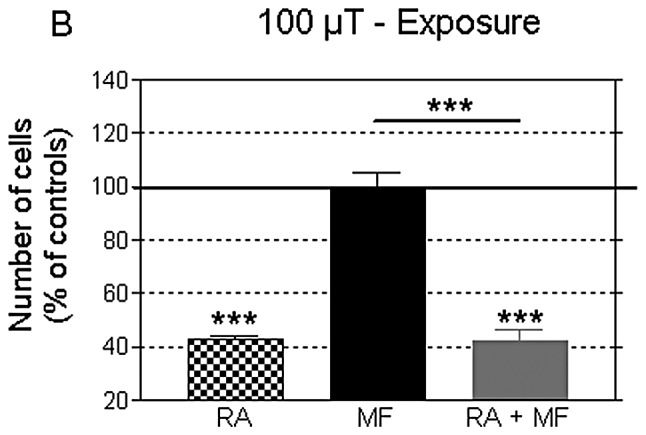
Cell number at the end of day 7 following the different testing conditions: controls (sham), RA, MF and combined treatment with RA+MF. The MF-exposed group was treated intermittently for 90 h, starting at day 3 post-plating. In the RA-treated groups the medium was supplemented with 2 μM RA at days 0 and 3 post-plating. (A) Exposure to MF at 10 μT; n=4 experimental replicates; 20 dishes per replicate and condition. (B) Exposure to MF at 100 μT; n=5 experimental replicates; 20 dishes per replicate and 25 dishes per condition. Data were normalized according to the values of the sham-exposed controls and expressed as the means ± SEM. ^***^P≤0.001 (ANOVA followed by unpaired Student’s t-test).

The data in Fig. 4 show that at day 7 post-plating, after 90 h of exposure, no changes were induced in the cell growth by any of the two flux densities tested. Thus, the proliferative response at 42 h of MF exposure was not observed when the treatment was prolonged for an additional 48-h period. In turn, on day 7 post-plating, the RA-induced decrease in cell number was more pronounced than that observed on day 5, both in the presence or in the absence of the MF. The cell viability was not significantly altered in any of the conditions tested (data not shown).

**Figure 5:**
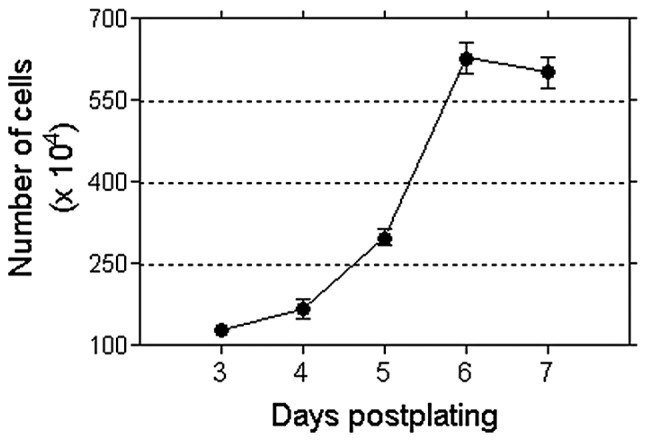
Growth pattern of NB69 cells cultured under control conditions. A decrease in cell number was observed at day 7, attributable to saturation by confluence and depletion of nutrients.

Concerning protein and DNA levels, the administration of 2 μM RA alone significantly reduced their levels, both at day 5 and 7 of treatment (Tables I and II), coincidentally with the observed reduction in cell number. Again, this response to RA was not affected by the simultaneous MF exposure for 42 or 90 h. Under most experimental conditions tested in the second series of experiments, treatment with MF alone did not change the total protein and DNA content. Exceptions to this was the DNA content at 42 h of exposure to MF at 10 μT (Table II) and the amount of protein at 90 h of treatment with 100 μT (Table I), which were modestly but significantly increased. This could indicate that an increase in the cell number may also occur at long-term exposures, after 90 h of MF stimulation starting at day 3 post-plating. However, the cell confluence in the cultures, almost reaching saturation at that time (Fig. 5) could have prevented the growth response to the MF. Experiments at a lower cell density would be needed to test this hypothesis. It should be noted that in this experimental series, the cell growth response induced by a 42-h exposure to MF at 100 μT was not found to be associated with incremental changes in DNA content (Table II), which is in contrast with the results obtained in the first series using the same flux density and exposure interval. This apparent discrepancy may be due to the relatively limited sensitivity of the spectrophotometric technique, which may fail to reveal differences in the DNA content when the differences in the cell number are in the rank of 10% or below.

### Immunocytochemical analysis of PCNA

**Figure 6:**
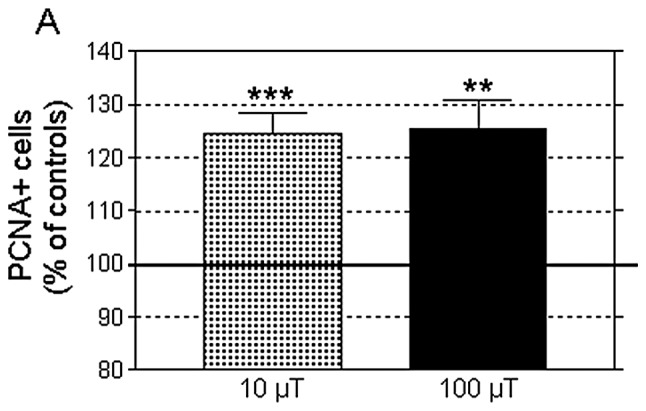

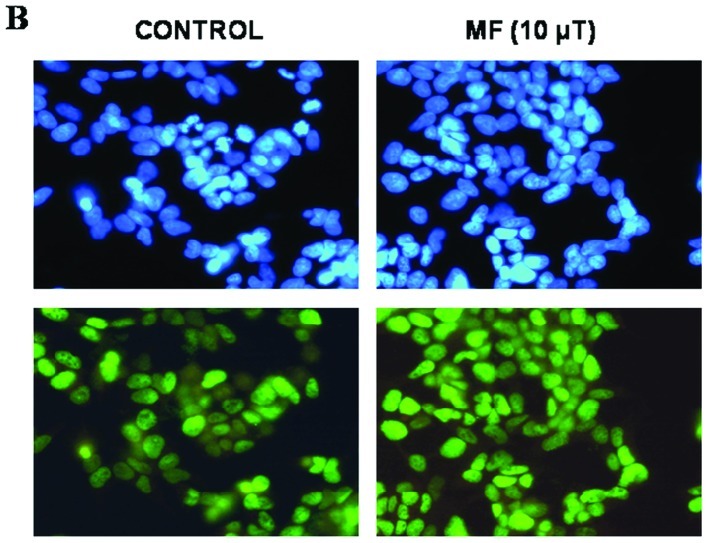
(A) PCNA-positive (PCNA^+^) cells at the end of day 5 in samples intermittently exposed to MF and/or incubated for 42 h. Data were normalized to sham-exposed controls and expressed as the means ± SEM of 8 and 3 experimental replicates, at 10 and 100 μT, respectively. ^**^P<0.01, ^***^P<0.001 (ANOVA followed by the Student’s t-test). (B) Representative example of immunostaining in controls and MF-exposed cells (10 μT). PCNA^+^ cells (lower panels) and Hoechst nuclear staining (upper panels). The PCNA^+^ rate was calculated as the number of cells expressing PCNA divided by the number of Hoechst-stained nuclei (total cell number).

**Figure 7:**
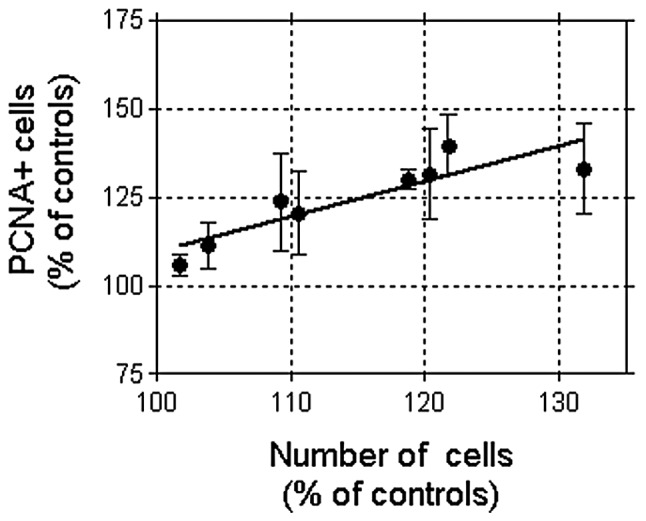
Linear correlation between the fraction of PCNA-positive cells and the total cell number, after 42 h of exposure to a 50-Hz MF at 10 μT. Each data point in the figure represents the mean ± SD of 8 dishes corresponding to each of 8 experimental replicates. Data were normalized to the corresponding sham-exposed controls (8 dishes per replicate). Pearson’s r=0.8832; P<0.01.

Assessment of the proliferative response to a 42-h exposure to MF at 10 or 100 μT was carried out through analysis of the levels of the proliferation marker PCNA at the end of the treatment. PCNA expression is known to be correlated with the proliferative activity in neuroblastomas (48). In addition, PCNA is necessary for nucleotide-excision repair of DNA (49). The analysis of PCNA expression revealed that both MF densities, 10 or 100 μT, significantly increased the proportion of PCNA-positive cells (24.5±4.07 and 25.6±5.27% over controls, respectively; Fig. 6), which confirmed the observed effects on cell growth. At MF at 10 μT the response was associated with an increased cell number (14.80±3.59% over the controls; P<0.01); both parameters, PCNA-positive cells and total cell number, being linearly correlated (8 experimental replicates, Pearson’s r=0.8832; P<0.01, Fig. 7). However, the number of replicates at 100 μT (n=3) was insufficient to provide a statistical correlation.

## Discussion

The present data showed that 42 h of intermittent exposure to a 50-Hz sinus wave MF induced similar cytoproliferative responses in the NB69 cell line at flux densities of 10 and 100 μT. On the other hand, 2 μM retinoic acid significantly reduced the cell number, as well as the protein and DNA contents at the different times assayed. This antiproliferative effect of RA was not significantly affected by simultaneous exposure to MF at 10 or 100 μT, indicating that RA inhibits the growth-promoting effects induced by an MF when administered alone.

Consistent evidence exists that power frequency MF can influence proliferation in different cell types when administered at densities of 1 mT or above. For instance, Delle Monache *et al*(50) reported that exposure to a sine wave 50-Hz MF at 1 mT increases proliferation in endothelial cells from human umbilical vein in a time-dependent manner, with statistically significant effects achieved at exposure periods of 6 h or longer. Vianale *et al*(18) also demonstrated that a 48-h exposure to a 50-Hz MF at 1 mT modulates keratinocyte growth through inhibition of the nuclear factor κ-light-chain-enhancer of activated B cell (NF-κB) signaling pathway. Wolf *et al*(51) reported that 24–72 h of exposure to 50-Hz MF at 0.5 to 1 mT, induced a dose-dependent increase in cell proliferation and in DNA damage (DNA strand breaks) in normal and cancer cells, including HL-60 leukemia cells, Rat-1 fibroblasts and WI-38 diploid fibroblasts. Proliferation of neuroblastoma cell lines was also found to be sensitive to ELF MF. For instance, 7 days of continuous exposure to a 50-Hz MF at 1 mT significantly increased, the proliferation rate by 10% in the neuroblastoma cell line LAN-5 (24). Exposure to a 50-Hz ELF-MF at 1 mT also induced cytoproliferation and dedifferentiation in SH-SY5Y neuroblastoma cells, triggering overexpression of proteins related to high malignant potential, drug resistance, cytoskeleton re-arrangement and enhanced defense against oxidative stress (52).

Besides of the above, a large number of studies have reported a heterogeneous variety of *in vitro* responses to MF (53–56) that, when taken together, have often been considered contradictory. However, at least part of such apparent contradictions can be attributable to the fact that the cellular response to ELF MF is dependent on a number of biological and physical parameters or factors, including the exposure time or the specific cell type used (57). For instance, the intermittency of exposure has recently been revealed as a critical factor in the proliferative response of NB69 cells to power frequency MF (47) which, in turn, supports the results and hypothesis of other authors (28,58).

Thus, the biological effects of weak ELF EMF, in particular those concerning promotion of cell proliferation, remain a matter of debate (reviewed in ref. 59). Among studies that have focused on the proliferative effects of power frequency MF, only a few have assayed magnetic flux density values below the safety levels recommended by ICNIRP for occupational protection against field-induced health effects (500 μT at f=50 Hz). In the present study, a proliferative effect was observed at the end of 42 h of intermittent exposure to weak magnetic flux densities of 10 and 100 μT, below the ICNIRP’s reference levels and much lower than those assayed in most of the studies described above. However, such a proliferative effect was not observable when the intermittent exposure was extended for an additional time duration of 48 h (total exposure time 90 h). At that time no differences were detected with respect to sham-exposed samples, except for a small, although statistically significant increase in total protein levels. In this regard, it must be taken into account that a spontaneous reduction in the cell number occurred in control samples at day 7 after seeding. This decline in cell proliferation may be attributable to the cell density-dependent regulation of growth, as well as to depletion of nutrients in the medium (60). Thus, in the event that the cell sensitivity to MF extended for periods longer than the 42 h of exposure, as indicated by the above mentioned increase in protein content, it would not be detectable in terms of changes in cell growth because of the described spontaneous decline in cell proliferation.

In order to identify the cellular events involved in the proliferative effects of an ELF MF, a number of studies have investigated the *in vitro* response to the fields when administered in combination with different chemical agents (61,62). Tonini *et al*(63) reported inhibition of proliferation in the neuroblastoma × glioma hybrid cell line NG108-15 when treated with the differentiating agent Bt2cAMP. This chemical inhibition of cell proliferation was counterbalanced by exposure to a 50-Hz MF at 120 or 240 μT. Retinoic acid is known to be one of the most potent inducers of differentiation in human neuroblastoma (64–66); however, the molecular mechanisms and signaling pathways that are responsible for RA-mediated neuroblastoma cell differentiation remain unclear. Retinoids are signaling molecules that are involved in cell proliferation, differentiation and apoptosis via both, non-receptor- and nuclear receptor-mediated pathways, thereby altering gene expression. Treatment of human neuroblastoma cell lines with retinoic acid causes a significant decrease in MYCN RNA expression and arrest of cell proliferation (65,67). The non-genomic effects of RA on neuroblastoma SH-SY5Y cells are mediated by the classical nuclear receptor, the retinoic acid receptor (RAR), which promotes activation of the PI3K and MAPK signaling pathways that intervene in RA-induced differentiation (68). The present results reveal that, administered at the physiological concentration of 2 μM, RA induces a significant decrease in cell number, associated with decreased protein and DNA contents, indicating that RA exerts antiproliferative effects in NB69 cells. These results support reports that RA induces specific phenotype expression and growth inhibition in a number of cell types, including NB69 (69–72). Our data also provide further support and additional rationale to the medical application of RA, alone or in combination with other chemicals, aimed to induce cell differentiation, apoptosis and growth arrest in tumors (37,67).

With regard to the cellular response to combined treatments with MF and RA, recent experimental data (73) showed that sinusoidal MF exposure (50 Hz, 1 mT), apart from enhancing the antiproliferative response induced by RA (5 μM), has a synergistic effect with RA-induced neural differentiation in the human neuroblastoma cell line BE(2)C. Moreover, Pirozzoli *et al*(24) found that RA induced an antiproliferative effect in the human neuroblastoma line LAN-5, which was significantly inhibited (by ~22%) by a 72-h exposure to a 50-Hz MF at 1 mT. In contrast to this, herein we report that a 42-h exposure to a 50-Hz MF at 10 or 100 μT did not inhibit or revert the antiproliferative effect nor the decrease in protein and DNA contents induced in NB69 cells by 2 μM RA. Rather, our data revealed that the presence of RA prevented or antagonized the proliferative effect induced by the MF. The differences between these 3 studies concerning the cellular response to RA in combination with an MF, could be due to the differences in the MF parameters and exposure protocols or in the RA concentrations tested. In addition, differences in the cellular genetics, including the varied presence of multiple copies of the MYCN oncogene and/or their transcriptional activation, which have been shown to be implicated in ELF MF responses (74) may be among the causes for the dissimilarities between these studies.

Moreover, we previously showed that the proliferative response of NB69 cells to a 50-Hz MF at 100 μT is mediated by the MAPK-ERK signaling pathway (47) that can elicit heterogeneous responses through cell type-specific regulatory mechanisms (75). Since the effects of RA on cell differentiation, proliferation and apoptosis are also mediated by MAPK-ERK (76), it is possible that RA can prevent the proliferative response to an MF by acting on the common ERK growth signaling pathway. The ERK pathway, also called the MEK-ERK cascade, is one of the main cytoplasmic signaling transduction systems that regulate processes of proliferation and survival in eukaryotic cells. Upregulation of ERK1/2 has been proposed to be implicated in tumor progression and metastasis in different cancer cell types (77,78). In the nervous system ERK1/2 has been connected to neuronal responses to stimuli, both functional (modulating neuronal survival, differentiation and plasticity) and pathological (such as Alzheimer’s or Parkinson’s disease) (79–81). Although the specific mechanism underlying the MF proliferative effect mediated by the MEK-ERK1/2 cascade is yet to be identified, it has been shown that ERK1/2 can be transiently activated by a variety of signals, including ELF MF (82) or UHF radio waves (83), as well as ionizing radiation (84).

Concerning DNA damage, several studies have reported increased DNA aberrations under specific MF exposure conditions (85,86). By contrast, others have not found such effects (87,88). Since it has been comfirmed that low frequency magnetic or electromagnetic fields do not transmit energy high enough to break chemical bonds, there is general agreement that these fields are unable of directly damaging DNA (89). Nevertheless, several hypotheses have been proposed on how electromagnetic fields might indirectly affect the structure of DNA (90–92). By reproducing experimental conditions described by Ivancsits *et al*(28) and using Comet assay analysis, Focke and coworkers (58) found a significant increase in DNA fragmentation in primary cultures of human fibroblasts exposed to a 50-Hz MF at 1 mT. The slight, although significant effect on DNA integrity was dependent on the intermittence of the exposure as well as on the cell line used; the latter indicating that the effect was mediated by biological factors of a genetic or physiological nature. These results also suggest that the potential genotoxic impact of the field may be due to slightly increased apoptosis and to disturbances in DNA transactions, both associated with the S-phase of the cell cycle. Although we do not know whether our exposure parameters induced DNA damage through a similar phenomenon, our data revealed that a 42-h exposure to an MF at 10 or 100 μT increased PCNA expression, which may be related to disturbances in the S-phase. Moreover, since a response was observed to be associated with an increased cell number, the present data indicate that the cytoproliferative effect of an MF may be mediated by stimulation of cell cycle progression in S-phase. This indication receives support from recently published data on increased BrdU incorporation in the DNA of NB69 cells intermittently exposed to a 50-Hz MF at 100 μT (34,47).

In conclusion, the herein reported results confirm previous indications that 42 h of intermittent exposure to a weak 50-Hz MF enhances proliferation in human neuroblastoma NB69 cells. The effects induced by two flux densities, 10 and 100 μT, were equivalent, non-significantly different from each other. The effects were manifested as a significant increase in the cell number. This increase was associated and linearly correlated to increased expression of PCNA, which was potentially related to the observed MF-induced disturbances in the S-phase of the cell cycle. If in fact these MF effects reflect an action exerted primarily on cell cycle-control molecules, this would reinforce prior data that the sensitive fraction of the cell population is the one undergoing cell division during the exposure intervals (34). The response to MF was fully inhibited or counteracted by 2 μM retinoic acid, which when administered alone or in combination with the MF, significantly reduced the cell number as well as the protein and DNA contents with respect to the corresponding untreated controls. This blocking of the MF effects may be exerted through RA-induced changes in the MAPK-ERK signaling pathway, which is known to intervene in the cellular response to both agents, RA and ELF MF (47,76). Collectively, the present data are of potential relevance to identify the mechanisms by which human cells are sensitive to ELF MF at flux densities below the reference levels recommended by ICNIRP (9,10) for protection against the deleterious effects of occupational or residential MF exposure.

## Figures and Tables

**Table I:** Changes in total protein content.

	Day 5 post-plating			Day 7 post-plating		
		
	RA	MF (42 h)	RA+MF	RA	MF (90 h)	RA+MF
10 μT	85.34±3.21^b^	110.10±6.56	83.56±3.56^b^	73.14±4.46^c^	95.75±3.09	72.53±1.55^c^
100 μT	76.81±5.33^b^	105.30±3.39	82.47±2.85^c^	63.66±4.50^c^	105.60±2.39^a^	68.44±7.52^b^

Total amount of protein (mg/dish) at the end of day 5 or 7 post-plating, following treatment with 2.0 μM RA and/or a 50-Hz MF, at 10 or 100 μT. Standardized data (%) over the respective controls, treated with vehicle and/or sham-exposed. Four and five experimental replicates were conducted at 10 or 100 μT, respectively. Each data value in the table represents the mean ± SEM of 20 dishes (at 10 μT) or 25 dishes (at 100 μT). ANOVA followed by the Student’s t-test;

a P≤0.05;

b P<0.01;

c P<0.001.

**Table II:** Changes in DNA content.

	Day 5 post-plating			Day 7 post-plating		
		
	RA	MF (42 h)	RA+MF	RA	MF (90 h)	RA+MF
10 μT	71.35±2.64^c^	124.8±9.83^a^	74.90±9.21^a^	60.45±7.61^b^	98.67±3.68	56.20±3.62^c^
100 μT	69.67±3.33^c^	98.33±3.74	71.06±2.28^c^	51.33±3.20^c^	96.43±3.98	48.35±2.37^c^

Total amount of DNA (mg/dish) at the end of day 5 or 7 post-plating, following treatment with 2.0 μM RA and/or a 50-Hz MF, at 10 or 100 μT. Standardized data (%) over the respective controls, treated with vehicle and/or sham-exposed. Four and five experimental replicates were conducted at 10 or 100 μT, respectively. Each data value in the table represents the mean ± SEM of 20 dishes (at 10 μT) or 25 dishes (at 100 μT). ANOVA followed by the Student’s t-test;

a P≤0.05;

b P<0.01;

c P<0.001.
